# Light-dependent chloroplast relocation in wild strawberry (*Fragaria vesca*)

**DOI:** 10.1080/15592324.2024.2342744

**Published:** 2024-04-17

**Authors:** Daisy Kiprono, Chonprakun Thagun, Yutaka Kodama

**Affiliations:** a Center for Bioscience Research and Education, Utsunomiya University, Tochigi, Japan; b Department of Biological Sciences, School of Pure and Applied Sciences, Meru University of Science and Technology, Meru, Kenya

**Keywords:** Blue light, chloroplast movement, *Fragaria vesca*, organelle, red light, woodland strawberry

## Abstract

Chloroplast photorelocation is a vital organellar response that optimizes photosynthesis in plants amid fluctuating environmental conditions. Chloroplasts exhibit an accumulation response, in which they move toward weak light to enhance photoreception, and an avoidance response, in which they move away from strong light to avoid photodamage. Although chloroplast photorelocation has been extensively studied in model plants such as *Arabidopsis thaliana*, little is known about this process in the economically important crop strawberry. Here, we investigated chloroplast photorelocation in leaf mesophyll cells of wild strawberry (*Fragaria vesca*), a diploid relative of commercially cultivated octoploid strawberry (*F*. × *ananassa*). Microscopy observation revealed that the periclinal area of leaf mesophyll cells in *F. vesca* is considerably smaller than that of *A. thaliana*. Given this small cell size, we investigated chloroplast photorelocation in *F. vesca* by measuring light transmittance in leaves. Weak blue light induced the accumulation response, whereas strong blue light induced the avoidance response. Unexpectedly, strong red light also induced the accumulation response in *F. vesca*. These findings shed light on chloroplast photorelocation as an intracellular response, laying the foundation for enhancing photosynthesis and productivity in *Fragaria*.

## Introduction

Chloroplasts relocate within plant cells in response to environmental stimuli such as light to optimize photosynthesis.^1^ For example, chloroplasts move toward low-intensity light to maximize photoreception (accumulation response)^2^ and move away from high-intensity light to reduce photodamage (avoidance response).^3^ These chloroplast photorelocation responses, which are conserved in many land plants, are induced by blue light (BL) through the BL receptor phototropin.^1^ An exception among land plants is the fern *Adiantum capillus-veneris*, whose accumulation response is induced not only by BL but also by red light (RL) via the unconventional chimeric BL and RL receptor neochrome and/or by photosynthesis.^4,5^ BL-induced chloroplast relocation enhances plant biomass in *Arabidopsis thaliana* ^2^ and alters leaf morphology in lettuce (*Lactuca sativa*).^6^ In flowering plants, chloroplasts also relocate to the bottom of the cell when plants are transferred from light to dark conditions (dark positioning).^1^ Further understanding chloroplast photorelocation could potentially help improve agronomic traits in various crops. However, to date, most studies on chloroplast photorelocation have been performed in model plant species such as *A. thaliana*.

Strawberry belongs to the economically important Rosaceae family, which includes apple, cherry, and peach. Wild strawberry (*Fragaria vesca*) is a diploid species related to commercially cultivated octoploid strawberry (*F*. × *ananassa*).^7^ Therefore, information obtained from analyzing the physiological responses and characteristics of *F. vesca* as a model *Fragaria* species could be used to improve the agronomic traits of *F*. × *ananassa*. To date, although various aspects of *F. vesca* have been studied (e.g., genomics, genetics, anatomy, and flowering),^8–11^ there has been little analysis of the cell biology or organelle biology of this species.

In this study, we characterized light-induced chloroplast relocation in *F. vesca* by measuring changes in light transmittance in leaves. Chloroplasts within leaf mesophyll cells of *F. vesca* generally exhibited an accumulation response under weak BL intensities, an avoidance response under strong BL intensities, and dark positioning when the plants were incubated in the dark. Unexpectedly, strong RL also induced the accumulation response of chloroplasts within plant cells. Our findings lay the foundation for future studies on chloroplast photorelocation and its underlying regulatory mechanism in *Fragaria* species.

## Materials and methods

### Plant materials and growth conditions

Wild strawberry (*Fragaria vesca* ‘Hawaii-4’; accession: PI551572)^11^ was cultivated and asexually maintained in a soil mixture under a 12-h/12-h light/dark photoperiod with 50 µmol photons m^−2^ s^−1^ of white fluorescent-light at 22°C. A healthy and vigorous mother plant with well-developed runners/stolons was chosen for propagation. Using a blade, runners/stolons were cut from the mother plant, leaving a stalk of 2 cm on the adaxial side and 1 cm on the abaxial side. Subsequently, these runners/stolons were transplanted to soil mixture (Super Mix A, Sakata Seed Co.), with the adaxial stalk embedded in the soil. To adapt the young plants to the new growing conditions, they were covered with transparent film for 3 days. One- or two-month-old plants were used in subsequent experiments.

### Microscopy

Images of cells and chloroplasts in *A. thaliana* and *F. vesca* were captured using a light microscope (BX60; Olympus) with a digital camera (DP73; Olympus). To compare the periclinal area of mesophyll cells in the two species, brightfield images of leaves from 1-month-old plants were obtained. The cell boundaries in the images were outlined with ImageJ software (https://imagej.net/ij/). Subsequently, the “area” measurement function within ImageJ was used to calculate the area enclosed by each cell. This process was repeated for all images obtained across four experiments, with measurements taken for five cells per experiment. Using the same images, the sizes of chloroplasts in the two species were compared by measuring the diameters of chloroplasts in five cells with ImageJ; the process was repeated four times.

### White-band assay

The white-band assay of *F. vesca* leaves was performed as described previously for *A. thaliana* .^12^ Briefly, fully expanded *F. vesca* leaves were cut off and placed adaxial side up on the surface of solidified 0.8% agar medium (0.8 g of agar powder in 100 mL of water). The leaves were covered with a transparent film and a slit board (2-mm-wide open slit), ensuring that the slit crossed the middle parts of the leaves. The leaves were exposed to strong BL (200 µmol photons m^−2^ s^−1^) from a blue LED at 470 nm (ISL-150 ×150-RHB; CCS Inc.) or RL (200 µmol photons m^−2^ s^−1^) from a red LED at 660 nm (ISL-150 ×150-RHB; CCS Inc.) for 2 h. Following exposure to the LEDs, the transparent film and plastic slit board were removed, and an RGB image of the leaves was captured using a scanner (GT-X970; EPSON).

To quantify the white band on the leaf, a blue channel image was isolated from an RGB image using ImageJ, and the light intensity at irradiated and non-irradiated areas was measured. The aspect ratio was obtained by calculating the intensity at the irradiated area divided by the intensity at the non-irradiated area. If no white band formed, the ratio was close to 1. However, if a white band formed, the ratio was < 1.

### Measuring leaf transmittance

Leaf transmittance was measured as described previously.^13^ Initially, the wells of a 96-well plate were filled with 300 µL of 1/4 Murashige and Skoog medium (FUJIFILM Wako Pure Chemical Co.) solidified with 0.5% (w/v) gellan gum (FUJIFILM Wako Pure Chemical Co.). Subsequently, a small leaflet (approximately 0.5 cm x 0.5 cm) was placed adaxial side up at the center of each well. The plate was then sealed with a transparent film to maintain high humidity and prevent sample desiccation, and two small holes per well were punched through the film using a toothpick to facilitate gas exchange. The plate was incubated in the dark for 24 h at 22°C to induce dark positioning. Note that the 24-h dark incubation period was confirmed to sufficiently induce the dark positioning (Supplementary Figure S1). Following incubation, light transmittance was measured using a microplate spectrophotometer (Spark10M; TECAN) with a wavelength of 660 nm. The sample plate was then irradiated with BL or RL of various intensities for 20 min (for each irradiation) using a blue or red LED, respectively. Light transmittance was measured again using the same measuring light at 660 nm in the spectrophotometer. To measure the change in light transmittance (ΔTransmittance (%)), light transmittance was measured before irradiation or any treatment (T_initial_) and again after irradiation treatment (T_final_). The final transmittance value was subtracted from the initial transmittance value to calculate the change in transmittance, which was expressed as ΔTransmittance = T_final_ − T_initial_. Finally, ΔTransmittance was converted to percentage and plotted.

### Statistical test

All statistical tests were performed using GraphPad Prism 10 (GraphPad Software Inc.).

## Results and discussion

### Comparison of periclinal area between *F.* *vesca* and *A.* *thaliana*

**Figure 1.**
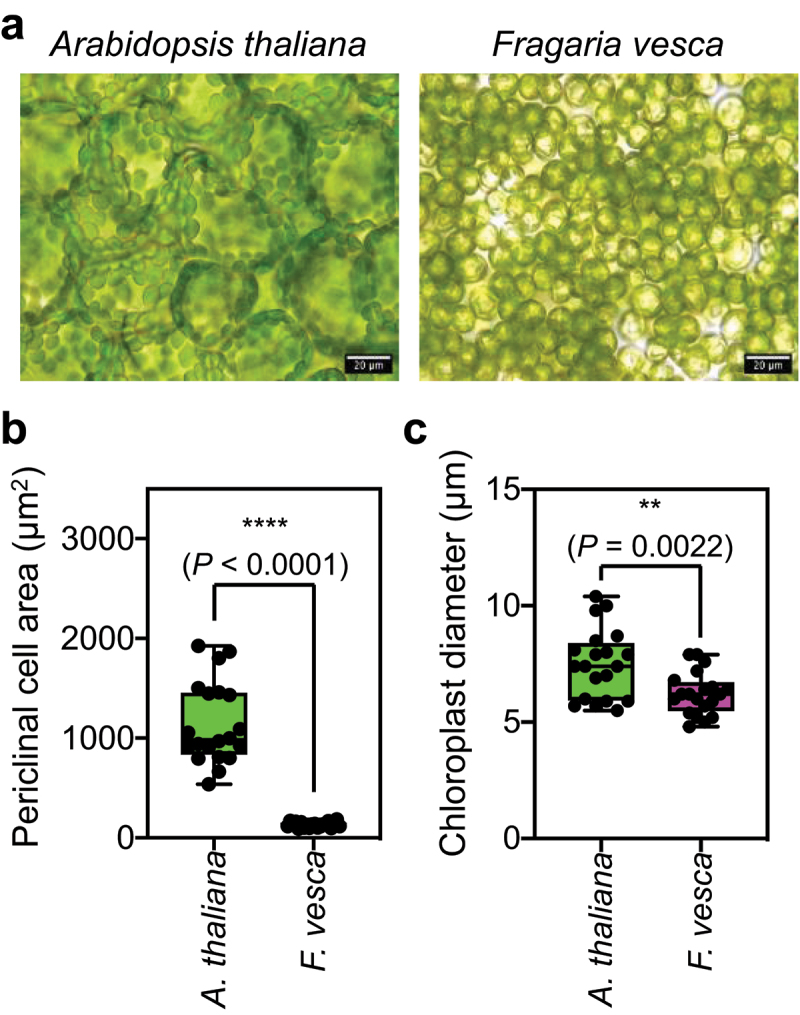
Leaf mesophyll cells of *F. vesca* are compact, with small periclinal areas.

Before analyzing chloroplast photorelocation, we initially observed the mesophyll cells on the adaxial sides of *F. vesca* leaves to measure cell size (periclinal area) and chloroplast size (diameter). The periclinal area of a leaf mesophyll cell in *F. vesca* was approximately 131 µm^2^, which is much smaller than that in *A. thaliana* (approximately 1,141 µm^2^) (Figure 1(a,b)). The diameters of chloroplasts in both plants were similar, although the chloroplasts were significantly smaller in *F. vesca* than in *A. thaliana* (Figure 1(a,c)).

(a) Representative brightfield images of leaf mesophyll cells of *A. thaliana* (left panel) and *F. vesca* (right panel). (b) Comparison of periclinal cell area between *A. thaliana* and *F. vesca* leaf mesophyll cells. (c) Comparison of chloroplast size (diameter) in *A. thaliana* and *F. vesca* leaf mesophyll cells. The periclinal cell areas and chloroplast sizes of five cells in one brightfield image were measured using ImageJ. The distributions of data from four independent experiments in (b) and (c) are presented as boxplots (*n* = 20). Upper and lower bars represent maximum and minimum values, respectively. Asterisks indicate significant differences, as analyzed by unpaired two-tailed Student’s t test; *P* values are shown.

Various techniques have been utilized to analyze chloroplast photorelocation, including microbeam-based single-cell analysis^14,15^ and leaf transmittance.^13,16^ The exceptionally small mesophyll cell size and congested periclinal region in *F. vesca* make it challenging to employ microbeam-based single-cell analysis to investigate chloroplast photorelocation in *F. vesca*. Therefore, we measured changes in leaf transmittance to investigate how chloroplast positioning changes under different light conditions.^13,16^

### Blue light – dependent chloroplast relocation in *F.* *vesca*

**Figure 2.**
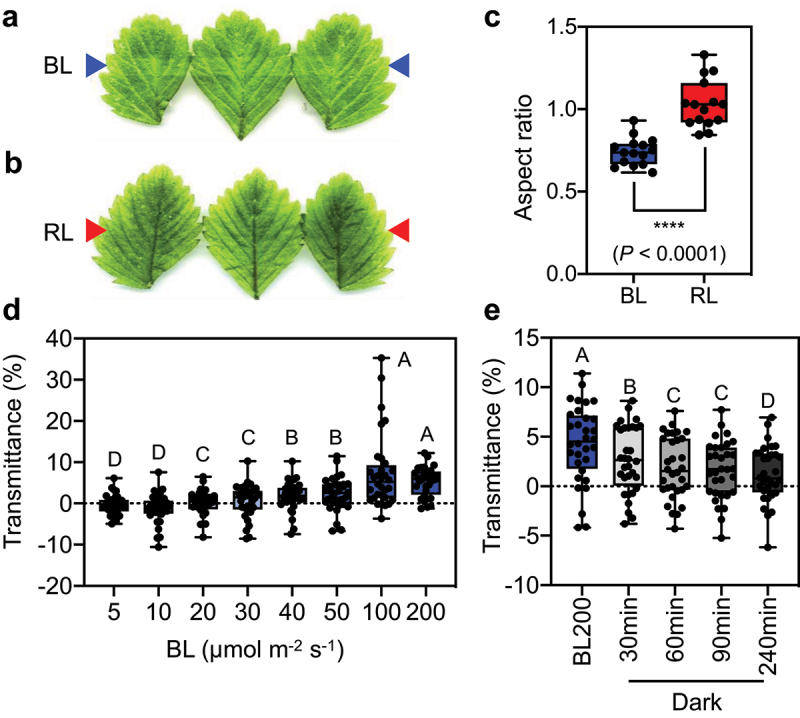
Blue light – dependent accumulation and avoidance responses in *F. vesca* (a) and (b) White-band assays of *F. vesca* leaves irradiated with 200 µmol photons m^−2^ s^−1^ of blue light (BL) at 470 nm (a) and red light (RL) at 660 nm (b). The images were manipulated to show clear white bands. Arrows indicate the positions of the irradiation bands. (c) Quantitative analysis of the white-band assay of *F. vesca* leaves. The distribution of aspect ratio values of five replicates from three biologically independent experiments (*n* = 15) is shown as a boxplot. Dots represent individual data points. Asterisks indicate significantly different mean aspect ratios, as analyzed by two-tailed unpaired Student’s t test. (d) Light-induced changes in transmittance in BL-treated leaf discs. *F. vesca* leaf discs were irradiated with various intensities of BL (5, 10, 20, 30, 40, 50, 100, and 200 µmol photons m^−2^ s^−1^) for 20 min. (e) Light transmittance in *F. vesca* leaf discs after different periods of dark incubation. In (d) and (e), light transmittance at 660 nm through the leaves was measured using a microplate spectrophotometer. Experimental data from four biologically independent experiments (eight replicates per experiment, *n* = 32) in (d) and (e) are shown as boxplots. Each dot in the boxplot represents the light transmittance in a replicate. Upper and lower bars represent maximum and minimum values, respectively. Different letters indicate significant differences in mean light transmittance among treatments, as analyzed by one-way ANOVA with Tukey’s HSD test at *p* = 0.05.

As a preliminary experiment, we performed a white-band assay, which is an easy way to measure leaf transmittance to analyze the avoidance response of chloroplasts in leaf cells under strong light conditions.^17^ A white band is observed due to the escape of chloroplasts from the periclinal areas of cells (*i.e*., avoidance response), resulting in increased light transmittance in leaves. We irradiated the middle parts of fully expanded *F. vesca* leaves with 200 µmol photons m^−2^ s^−1^ of BL for 2 h. Exposing leaves to this strong BL intensity caused clear white bands to appear on the leaves, indicating that the avoidance response of chloroplasts occurred within *F. vesca* leaf mesophyll cells (Figure 2(a)). In contrast, we observed no change in leaf color when the middle parts of leaves were irradiated with 200 µmol photons m^−2^ s^−1^ of RL (Figure 2(b)). Quantitative analysis of the results of the white-band assay revealed that high-intensity BL induced a significantly more pronounced chloroplast avoidance response in leaf mesophyll cells compared to high-intensity RL (Figure 2(c)). These results suggest that chloroplast photorelocation within *F. vesca* in leaf mesophyll cells is more dependent on changes in BL rather than RL. However, further study is needed to understand the distinct responses of chloroplasts within *F. vesca* leaf cells to various light intensities.

To investigate the level of BL required to induce the accumulation and avoidance responses, we sequentially exposed the samples to various BL intensities (5, 10, 20, 30, 40, 50, 100, and 200 µmol photons m^−2^ s^−1^) for 20 min after dark adaptation. We quantified leaf transmittance using the microplate reader-based method employed in a previous study.^13^ Changes in leaf transmittance are indicative of chloroplast responses. A decrease in transmittance indicates the accumulation response (ΔTransmittance (%) is lower than zero), whereas an increase in transmittance indicates the avoidance response of chloroplasts in leaf mesophyll cells.^13,16^ Our analysis of leaf transmittance revealed the occurrence of the chloroplast accumulation response in *F. vesca* leaf mesophyll cells under 5 and 10 µmol photons m^−2^ s^−1^ of BL treatment (Figure 2(d)). However, the medians of ΔTransmittance (%) significantly increased in leaf discs subsequently irradiated with 20, 30, 40, 50, 100, and 200 µmol photons m^−2^ s^−1^ of BL, indicating that the chloroplasts in leaf cells exhibit the avoidance response at these BL intensities (Figure 2(d)). The threshold for BL intensities that induced changes in chloroplast relocation behaviors within plant cells appears to be within the range of 10 to 20 µmol photons m^−2^ s^−1^ (Figure 2(d)).

The photorelocation of chloroplasts in plant cells is a reversible biological process that protects plants from stress caused by strong light intensity and enhances photosynthesis. Chloroplasts reposition themselves to the bottoms of cells when plants are moved from light to dark conditions (*i.e*., dark positioning). We demonstrated that high BL intensities (up to 200 µmol photons m^−2^ s^−1^) increased the chloroplast avoidance response in *F. vesca* mesophyll cells (Figure 2(d)). We then transferred leaves that had been irradiated with 200 µmol photons m^−2^ s^−1^ of BL to the dark. We observed a significant decline in the median ΔTransmittance (%) over time after transfer to the dark (Figure 2(e)). This result suggests that the positions of chloroplasts within *F. vesca* leaf mesophyll cells also change during the transition from light to dark conditions.

The finding that chloroplast photorelocation in *F. vesca* leaf mesophyll cells is dependent on BL is consistent with many previous reports in various plant species.^13,14,18^ We reasoned that the photoreceptor phototropin likely mediates the BL-dependent chloroplast relocation and photoresponse in *F. vesca*. Upon querying the *PHOTOTROPIN* genes in the *F. vesca* genome database (F.vesca_v2.0.a1) of Strawberry GARDEN (https://strawberry-garden.kazusa.or.jp/index.html),^19^ we identified gene13896-v1.0-hybrid as *PHOTOTROPIN1* and gene17125-v1.0-hybrid as *PHOTOTROPIN2*. In the future, we plan to combine our leaf transmittance analysis with biotechnological methods such as genome editing to study the underlying mechanism of chloroplast photorelocation in *F. vesca*.

### The accumulation response is induced under strong RL conditions

**Figure 3.**
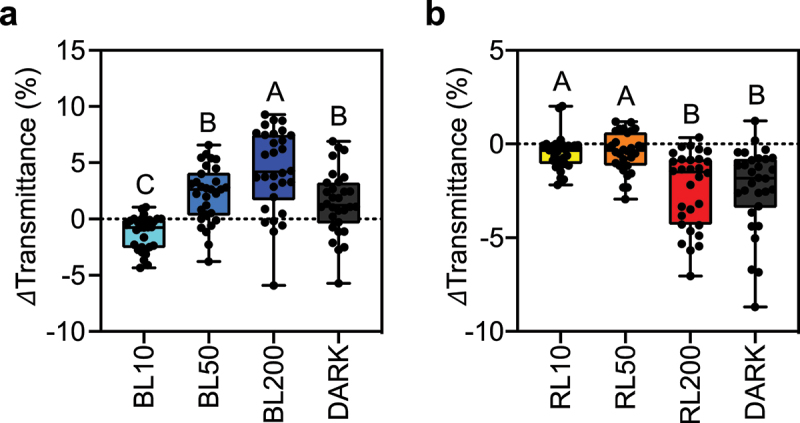
Comparison of blue- and red-light responses (a) Blue light (BL) – dependent changes in leaf transmittance. (b) Changes in light transmittance in red light (RL) – irradiated leaves. Leaf discs dissected from dark-acclimated *F. vesca* leaves were irradiated with BL or RL at 10, 50, and 200 µmol photons m^−2^ s^−1^ to study the effects of light sources on chloroplast photorelocation within the mesophyll cells of *F. vesca* leaves. Following strong light irradiation at 200 µmol photons m^−2^ s^−1^, the leaf discs were transferred to a dark chamber and incubated for 24 h before measuring light transmittance using a microplate reader. Thirty experimental data points from four biologically independent experiments (*n* = 32) are shown as boxplots. Each dot represents an experimental value. Upper and lower bars in the boxplots indicate the maximum and minimum values, respectively. Different letters indicate significant differences in mean light transmittance values among treatment groups, as analyzed by one-way ANOVA with Tukey’s HSD test at *p* = 0.05.

To more precisely examine chloroplast photorelocation in *F. vesca*, we developed a highly reproducible method based on the BL intensities highlighted in Figure 2(d). This method utilizes two distinct light sources (BL and RL) across a range of light intensities to investigate the BL dependency in inducing chloroplast relocation within leaf mesophyll cells (Figure 3). After 24 h of dark adaptation, we induced the accumulation response by treating leaf disks with 10 µmol photons m^−2^ s^−1^ of BL. The avoidance response was induced by 50 µmol photons m^−2^ s^−1^ of BL, and a significantly stronger avoidance response was induced at 200 µmol photons m^−2^ s^−1^ of BL (Figure 3(a)). The chloroplasts in plant cells exhibited dark positioning after the plant materials were subsequently incubated in the dark (Figure 3(a)).

We irradiated *F. vesca* leaves with various RL intensities (10, 50, and 200 µmol photons m^−2^ s^−1^) to explore the responses of chloroplasts to RL. The chloroplasts exhibited no significant changes in location within cells when treated with 10 or 50 µmol photons m^−2^ s^−1^ of RL (Figure 3(b)). Surprisingly, a notable decrease in leaf transmittance was observed under strong RL conditions (200 µmol photons m^−2^ s^−1^) and after dark treatment (Figure 3(b)). These results suggest that *F. vesca* not only has conventional BL-dependent chloroplast relocation responses but also has a strong RL-induced accumulation response.

The strong RL-induced accumulation response in *F. vesca* was unexpected (Figure 3(b)) because when the same light intensity was used in the band assay, no accumulation response-mediated green band^19^ was observed (Figure 2(b)). One explanation for this discrepancy may be that leaf transmittance analysis using a microplate spectrophotometer is a more sensitive method than the band assay. A RL-induced accumulation response has been reported in the fern *A. capillus-veneris*, which is induced by neochrome and/or photosynthesis.^4,5^ However, no gene similar to *NEOCHROME* was found within the *F. vesca* genome database (F.vesca_v2.0.a1). The robust RL-induced accumulation response of chloroplasts within leaf mesophyll cells in *F. vesca* might be induced by photosynthesis. The physiological significance of the RL-induced accumulation response in *F. vesca* remains to be determined. Further study is needed to understand this phenomenon in *F. vesca*.

### Concluding remarks

*F. vesca*, a diploid model species closely related to the commercially cultivated octoploid strawberry *F. × ananassa* ,^7^ plays a crucial role in strawberry research. Our study unveiled the influence of light on the precise control of chloroplast positioning within the mesophyll cells of *F. vesca* leaves. Given that chloroplast positioning plays a pivotal role in the photosynthesis, biomass production, and overall physiological characteristics of plants, our findings have significant implications for improving the agronomic traits of *Fragaria* species, thereby enhancing the traits of commercially cultivated strawberries such as *F*. × *ananassa*.

## Supplementary Material

Supplemental Material

## Data Availability

All data are available in the manuscript.
