# Supplementary material for: Light-dependent chloroplast relocation in wild strawberry (Fragaria vesca)
Source: Plant Signal Behav. 2024 Apr 17;19(1):2342744. doi: 10.1080/15592324.2024.2342744 (PMC11028000; doi:10.1080/15592324.2024.2342744)
Supplement: Supplemental Material [file KPSB_A_2342744_SM5719.docx]

**Supplementary Figure S1. Leaf transmittance of *F. vesca* leaves under dark incubation**

(**a**) Leaf discs of *F. vesca* were incubated under dark conditions after pre-incubation under the standard culture conditions (a 12-h/12-h light/dark photoperiod with 50 µmol photons m^−2^ s^−1^ of white fluorescent-light at 22°C). Changes in chloroplast positioning within plant cells were analyzed through light transmittance analysis before (pre-incubation during the light period) and after incubating plant samples in darkness for 24 and 48 hours. Arrows indicate the time points of light transmittance analysis. (**b**) Light transmittance analysis of *F. vesca* leaves cultured in darkness. Twenty-four experimental datasets from three biologically independent experiments (*n* = 24) are presented as boxplots. Each dot represents an individual experimental value. The upper and lower bars in the boxplots indicate the maximum and minimum values, respectively. The black bars within the boxplots represent the medians of light transmittance values at each time point. Different letters indicate statistically significant differences in mean light transmittance among treatment groups, as determined by ANOVA with Tukey’s HSD test at *p* = 0.05.
